# Prevalence of periodontitis and associated factors among Portuguese Air Force pilot cadets: a cross-sectional study

**DOI:** 10.3389/fpubh.2025.1724111

**Published:** 2025-12-03

**Authors:** Diana Martins, João Botelho, André Júdice, Luís Proença, José João Mendes, Vanessa Machado

**Affiliations:** Egas Moniz Center for Interdisciplinary Research (CiiEM), Egas Moniz School of Health & Science, Almada, Portugal

**Keywords:** periodontal disease, military personnel, Portuguese Air Force, aviation occupational health, oral health-related quality of life, oral health values

## Abstract

**Objectives:**

To assess the periodontal health, perceived quality of life, and oral health values among Portuguese Air Force (PT-AF) pilot cadets.

**Methods:**

This cross-sectional study included cadets from the PT-AF Academy (December 2024–Mar 2025). Participants completed sociodemographic, behavioral, Oral Health Impact Profile-14 (OHIP-14) and Oral Health Values Scale-12 (OHVS-12) questionnaires, followed by full-mouth periodontal examinations to compute Periodontal Inflamed Surface Area (PISA) and Periodontal Epithelial Surface Area (PESA), and classify periodontal status according to the 2017 European Federation of Periodontology/American Academy of Periodontology (EFP/AAP) criteria. Descriptive and inferential analyses were conducted, with a significance level set at *p* ≤ 0.05.

**Results:**

Among 90 participants (72% of the contingent; mean age 21.1 ± 1.7 years), 22.2% [95% confidence interval (CI): 14.6–31.6] had periodontitis and 22.2% (95% CI: 14.6–31.6) gingivitis. Only 34.4% reported regular interproximal cleaning. Periodontitis was significantly associated with smoking habits (*p* = 0.029) and was more prevalent among 5th-year cadets (*p* = 0.002). No significant differences were found in OHIP-14 or OHVS-12 scores by periodontal status, except for the “Appearance and Health” OHVS-12 domain, where those with periodontitis scored higher (*p* = 0 034). Age correlated positively with PISA (*p* = 0.003).

**Conclusions:**

Despite their young age and military fitness, nearly half of PT-AF cadets presented clinical signs of periodontal disease. The findings underscore the importance of structured and integrating oral health surveillance and preventive strategies within military occupation health programs.

## 1 Introduction

Periodontitis is a chronic inflammatory disease affecting the supporting tissues of the teeth, with a global prevalence of 62% among adults aged 18 years and older (1, 2). Beyond local oral destruction and potential tooth loss, this condition is closely linked to systemic diseases (3) and is associated with reduced chewing function and aesthetics, posing a burden to overall health and quality of life (4, 5). In military aviation, chewing impairment and aesthetic concerns may affect nutritional intake, communication during flights, and consequently operational readiness (6). Also, the pathogenesis is largely associated with dysbiotic dental plaque biofilms and is driven by modifiable risk factors, including poor oral hygiene, smoking habits, and psychological stress (7, 8).

The aviation environment subjects aircrew to extreme barometric fluctuations, particularly during ascent and descent. These pressure variation may lead to barodontalgia, an acute dental pain caused by trapped air within teeth or periodontal pockets (7, 9). Although often underreported, barodontologia has been documented in 5.4% of military aircrew (10). Also, pilot cadets are exposed to aviation-specific stressors such as G-forces, hypoxia and circadian disruption, which affect physiological and cognitive performance. Exposure to high G-forces alters hemodynamics and perception, hypoxia impairs reaction time and attention, and irregular schedules contribute to fatigue and reduced alertness (11–13). These occupational exposures, coupled with behavioral risk factors, impair performance, and compromise flight safety and mission effectiveness (7, 9).

According to the European Military Medical Service, nearly 10% of oral emergencies during deployment and maneuvers are attributed to periodontal disease, often leading to temporary disqualification from service, compromising the operability of the unit (14, 15), In North Atlantic Treaty Organization (NATO) countries, up to 14% of enlisted Dutch military personnel are considered dentally unfit for deployment due to oral health conditions, and enlisted ranks display significantly higher caries experience and poorer oral hygiene than officers and non-commissioned officers (16). In the United States Air Force, over 35% of incoming recruits require urgent or extensive dental treatment, mainly due to untreated dental caries and periodontitis (17). Additionally, smoking prevalence among military personnel can reach 35% among lower-ranked Dutch military personnel (16), further compounding oral health risks. NATO has recognized this issue by adopting standardized dental fitness classifications for deployment eligibility.

Given these unique occupational hazards, the oral health of aircrew personnel must be closely monitored and preserved. In Portugal, the Portuguese Air Force (PT-AF) has implemented structured training programs through the Air Force Academy (AFA) to prepare pilot officers (“Pilotos Aviadores”—PILAV) for operational roles (18). Despite access to military dental services, with Military Dentists and a proper number to provide services across internal Health Units, the lack of a centralized oral health strategy persists (19). National data reveal suboptimal oral health literacy and high smoking rates among military subgroups, with tobacco use reaching 45.9% and caries prevalence exceeding 50.0% (20, 21). However, specific data focusing on periodontal health among PT-AF personnel remains scarce.

Given the operational demands placed on pilot officers and their role in national defense and international missions, assessing their periodontal health is critical. Therefore, this study aimed to characterize the periodontal health status of PT-AF pilot cadets, explore potential associations with behaviors and risk factors, and support the development of targeted preventive strategies within military oral healthcare.

## 2 Materials and methods

### 2.1 Study design and setting

This cross-sectional study was conducted at the Portuguese Air Force Academy, a key institution for the academic and professional training of future military pilots. The study complied with the Declaration of Helsinki of 1975, as revised in 2024, and was approved by both the Air Force Ethics Committee (approval number: SAI_FAP_2024_3918) and the Ethics Committee of Egas Moniz (approval number: PT-199/24). Eligible pilot cadets were invited to voluntarily and anonymously participate. Written informed consent was obtained from each participant prior to inclusion in the study. The results are reported based on the STrengthening the Reporting of OBservational studies in Epidemiology (STROBE) guidelines (22).

### 2.2 Participants and study size

The inclusion criteria required participants to be actively enrolled in the Portuguese Air Force Pilot Officer Training program (from the 1st to the 5th year), be 18 years of age or older, and provide written informed consent. Individuals with systemic conditions that contraindicate periodontal examination were excluded. Within the Portuguese Air Force training structure, cadets begin flight training activities in the 1st academic year.

The inclusion of participants accounted for a consecutive convenience sampling of all pilot cadets undergoing training at AFA (*n* = 125), over a 4-month timeframe, from December 2024 to March 2025. During this period, a representative sample of 90 were enrolled across all academic years, resulting in a participation rate of 72.0%. Participation by year was as follows: 5th year–30/30 (100%); 4th year–11/18 (61.1%); 3rd year–21/23 (91.3%); 2nd year–18/28 (64.3%); and 1st year–10/26 (38.5%).

### 2.3 Variables, data sources and measurement

Sociodemographic and behavioral data was collected through a structured, self-administered questionnaire. Information included age, sex, nationality, race, marital status (categorized as married/in a union, divorced, single, or widowed), and education level, classified according to the 2011 International Standard Classification of Education (ISCED-2011) as Middle (ISCED 3–4) and Higher (ISCED 5–8) (23). Occupation status was defined as being a PILAV student (categorized by academic year, from 1st to 5th). Behavioral data included smoking habits (non-smoker, former smoker, or current smoker) and oral hygiene practices, including the frequency of daily toothbrushing and methods of interproximal cleaning.

Participants also completed the validated Portuguese self-reported versions of the Oral Health Impact Profile (OHIP)-14 (24) and Oral Health Value Scale (OHVS)-12 (25) tools. The OHIP-14 assesses the perceived impact of oral health on daily life across seven domains (functional limitation, physical pain, psychological discomfort, physical disability, psychological disability, social disability, and handicap), with 14 items rated on 5-point Likert scale (0 = never to 4 = almost always). Total scores range from 0 to 56, with higher scores indicating worse oral health-related quality of life (OHRQoL). The OHVS-12 evaluates oral health values across four domains—professional dental care, appearance and health, flossing, and maintaining natural teeth—using a 5-point Likert scale (1 = strongly disagree to 5 = strongly agree), where higher scores reflect more positive oral health values (26).

A comprehensive periodontal examination was performed by a calibrated examiner at the Health Unit of Air Base No. 1 (Sintra, Portugal). Assessments were conducted under standard clinical conditions, with participants examined on an adjustable stretcher under adequate lighting. Radiographs were not taken due to ethical restrictions regarding unnecessary radiation exposure. All fully erupted teeth—excluding third molars, implants, and retained roots—were assessed using a sterilized dental mirror and a North Carolina periodontal probe-15 (Hu-Friedy, Chicago, IL, USA). The following clinical parameters recorded at six sites per tooth (mesiobuccal, buccal, distobuccal, mesiolingual, lingual, and distolingual): plaque index (27), gingival index, bleeding on probing (BoP), probing depth (PD), gingival recession (REC), and the number of missing teeth. PD was determined as the distance from the free gingival margin to the pocket's base, while REC was the distance from the cementoenamel junction (CEJ) to the free gingival margin, with a negative sign assigned if the gingival margin was above the CEJ. CAL was calculated as the algebraic sum of REC and PD for each site, with measurements rounded down to the nearest whole millimeter.

Periodontal health status was classified as healthy, gingivitis, or periodontitis according to the 2017 European Federation of Periodontology/American Academy of Periodontology (EFP/AAP) case definition (8, 28). After the examination, participants received individual feedback and an educational leaflet on gingival inflammation, prevention, and treatment options.

The periodontal inflamed surface area (PISA) and periodontal epithelial surface area (PESA) were subsequently calculated per tooth using Microsoft Excel (29, 30). First, mean CAL and REC were determined for each tooth. PESA was derived from these values using standard formulas; PISA was then calculated by multiplying PESA by the proportion of BoP-positive sites. Total PISA and PESA values were expressed in mm^2^ as the sum of all teeth per participant.

### 2.4 Measurement reliability and reproducibility

Examiner calibration ensured measurement reliability. One clinician was trained under a periodontist's supervision. Calibration was performed in 10 volunteers (not included in study sample). PD and CAL were recorded twice, 1 week apart. Intraclass correlation coefficients showed excellent reliability (inter-examiner ICC = 0.97 for CAL and 0.96 for PPD; intra-examiner ICC = 0.96–0.98 for both parameters).

### 2.5 Statistical methods

All statistical analyses were conducted using the IBM SPSS Statistics version 29.0 (Armonk, New York, USA). Data were submitted to descriptive and inferential analysis methodologies. Descriptive statistics were summarized and presented as mean and standard deviation (SD). Categorical variables were summarized using frequencies and percentages. Differences in clinical and behavioral characteristics across periodontal status groups (healthy, gingivitis, periodontitis) were evaluated using the Kruskal–Wallis test for continuous non-normally distributed variables and the Chi-squared test for categorical variables. Spearman's rank correlation coefficient (rho) was employed to assess correlations among quantitative variables, including clinical periodontal markers, quality of life, and oral health values. A significance level of 5% (*p* ≤ 0.05) was considered for all inferential analyses.

## 3 Results

### 3.1 Participants and descriptive data

A total of 90 pilot cadets participated in this study, representing 72% of the total eligible cohort. The mean age was 21.1 (±1.7) years. Overall, all participants were Caucasians and single, and the vast majority were male (95.6%, *n* = 86), with only four female participants (5.4%). Regarding smoking status, most trainees were not active smokers (38.9% former smokers, and 44.4% non-smokers), with 16.7% of current smokers. Most cadets (87.7%) reported brushing their teeth at least twice daily, while 12.2% reported brushing only once per day. Interproximal cleaning was performed regularly by 34.4%, occasionally by 20.0%, and not at all by 45.6% of participants (Table 1).

**Table 1:** Sociodemographic characteristics and behaviors toward oral health of the included participants, presented as *n* (%), according to the periodontal status (*N* = 90).

	**Periodontal status**					**Total (*****n*** = **90)**
	**Healthy (*****n*** = **50)**	**Gingivitis (*****n*** = **20)**	**Periodontitis (*****n*** = **20)**			
			**Stage I (*****n*** = **10)**	**Stage II (*****n*** = **9)**	**Stage III (*****n*** = **1)**	
**Sex**, ***n*** **(%)**						
Male	47 (94.0)	19 (95.0)	10 (100.0)	9 (100.0)	1 (100.0)	86 (95.6)
Female	3 (6.0)	1 (5.0)	0 (0.0)	0 (0.0)	0 (0.0)	4 (4.4)
Age (years), mean (SD)	20.6 (1.7)	21.4 (1.8)	21.8 (1.5)	22.2 (1.7)	21 (NA)	21.1 (1.7)
**Smoking status**, ***n*** **(%)**						
Current smoker	5 (10.0)	6 (30.0)	3 (30.0)	1 (11.1)	0 (0.0)	15 (16.7)
Former smoker	17 (34.0)	11 (55.0)	3 (30.0)	4 (44.4)	0 (0.0)	35 (38.9)
Non smoker	28 (56.0)	3 (15.0)	4 (40.0)	4 (44.4)	1 (100.0)	40 (44.4)
**Education level**, ***n*** **(%)**						
Middle	24 (48.0)	9 (45.0)	6 (60.0)	6 (66.7)	0 (0.0)	45 (50.0)
Higher	26 (52.0)	11 (55.0)	4 (40.0)	3 (33.3)	1 (100.0)	45 (50.0)
**Academic year**, ***n*** **(%)**						
1st	6 (12.0)	2 (10.0)	1 (10.0)	1 (11.1)	0 (0.0)	10 (11.1)
2nd	13 (26.0)	3 (15.0)	1 (10.0)	1 (11.1)	0 (0.0)	18 (20.0)
3rd	11 (22.0)	7 (35.0)	1 (10.0)	1 (11.1)	1 (100.0)	21 (23.3)
4th	7 (14.0)	3 (15.0)	1 (10.0)	0 (0.0)	0 (0.0)	11 (12.2)
5th	13 (26.0)	5 (25.0)	6 (60.0)	6 (66.7)	0 (0.0)	30 (33.3)
**Brushing frequency (daily)**, ***n*** **(%)**						
1	7 (14.0)	1 (5.0)	3 (30.0)	0 (0.0)	0 (0.0)	11 (12.2)
2	30 (60.0)	17 (85.0)	3 (30.0)	7 (77.8)	0 (0.0)	57 (63.3)
3+	13 (26.0)	2 (10.0)	4 (40.0)	2 (22.2)	1 (100.0)	22 (24.4)
**Interproximal cleaning**, ***n*** **(%)**						
Yes	21 (42.0)	4 (20.0)	4 (40.0)	2 (22.2)	0 (0.0)	31 (34.4)
Occasionally	7 (14.0)	6 (30.0)	1 (10.0)	4 (44.4)	0 (0.0)	18 (20.0)
No	22 (44.0)	10 (50.0)	5 (50.0)	3 (33.3)	1 (100.0)	41 (45.6)

SD, standard deviation.

### 3.2 Periodontal status

The majority was diagnosed as periodontally healthy (55.6%, 95% CI: 45.2–65.5, *n* = 50), 20 with gingivitis (22.2%, 95% CI: 14.6–31.6), and 20 with periodontitis (22.2%, 95% CI: 14.6–31.6). Of those with periodontitis, 10 (11.1%) were classified as Stage I, 9 (10.0%) as Stage II, and 1 participant (1.1%) as Stage III according to the 2018 EFP/AAP classification (Table 1 and Supplementary Table 1). All women were diagnosed with periodontal health or gingivitis.

Periodontitis was significantly more frequent among 5th-year cadets (60% of periodontitis cases), while the majority of healthy and gingivitis cases were found in earlier years [1st−4th year; χ^2^ (2, *N* = 90) = 8.235, *p* = 0.002]. Smoking status was significantly associated with periodontal condition [χ^2^ (4, *N* = 90) = 10.725, *p* = 0.029]. A clear trend was observed in smoking status across groups: the prevalence of current smokers was higher among individuals with gingivitis (30.0%) and periodontitis (20.0%) compared to the healthy group (10.0%; Table 2, Supplementary Table 2).

**Table 2:** Participants distribution across academic years and smoking status as a function of their periodontal status (*N* = 90).

	**Healthy (*n* = 50)**	**Gingivitis (*n* = 20)**	**Periodontitis(*n* = 20)**	*p*-value^*^
**Academic year**, ***n*** **(%)**				
1–4th years	37 (74.0)	15 (75.0)	8 (40.0)	0.002
5th year	13 (26.0)	5 (25.0)	12 (60.0)	
**Smoking status**, ***n*** **(%)**				
Current smoker	5 (10.0)	6 (30.0)	4 (20.0)	0.029
Former smoker	17 (34.0)	11 (55.0)	7 (35.0)	
Non smoker	28 (56.0)	3 (15.0)	9 (45.0)	

^*^Chi-squared test.

Regarding oral hygiene, daily brushing frequency of three or more times was more common among participants with periodontitis (40.0%) than in those with gingivitis (10.0%) or healthy status (26.0%). However, regular interproximal cleaning was most frequent in the healthy group (42.0%) and least frequent in those with Stage III periodontitis (0.0%; Table 1).

### 3.3 Quality of life and oral health values and periodontal status

The analysis of OHIP-14 scores revealed no statistically significant differences between periodontal health groups across total or domain-specific scores (all cases *p* > 0.05). The mean total OHIP-14 score was 2.9 (±3.3) for healthy participants, 3.5 (±5.0) for those with gingivitis, and 3.2 (±5.8) for those with periodontitis (*p* = 0.461; Table 3 and Supplementary Table 3).

**Table 3:** Distribution of OHIP-14 and OHVS-12 scores (presented as mean, standard deviation and 95% confidence interval for mean), as a function of periodontal status.

**Variable**	**Healthy**	**Gingivitis**	**Periodontitis**	*p*-value^*^	**Total**
**OHIP-14**					
Total	2.9 (3.3) [2–3.8]	3.5 (5) [1.2–5.9]	3.2 (5.8) [0.6–5.9]	0.461	3.1 (4.3) [2.2–4.0]
Functional limitation	0.2 (0.6) [0.1–0.4]	0.3 (0.8) [0–0.7]	0.1 (0.5) [-0.1–0.4]	0.667	0.2 (0.6) [0.1–0.4]
Physical pain	1.1 (1.1) [0.8–1.4]	1.4 (1.9) [0.4–2.3]	1.4 (2) [0.4–2.3]	0.913	1.2 (1.5) [0.9–1.5]
Psychological discomfort	0.5 (0.9) [0.3–0.8]	0.7 (1.1) [0.2–1.2]	0.4 (1) [0–0.9]	0.539	0.6 (1.0) [0.4–0.8]
Physical disability	0.3 (0.8) [0.1–0.6]	0.4 (0.9) [0–0.9]	0.6 (1.1) [0–1.1]	0.801	0.4 (0.9) [0.2–0.6]
Psychological disability	0.4 (0.8) [0.1–0.6]	0.4 (0.8) [0–0.8]	0.3 (0.7) [0–0.6]	0.798	0.4 (0.7) [0.2–0.5]
Social disability	0.2 (0.6) [0–0.4]	0.1 (0.5) [-0.1–0.4]	0.2 (0.5) [0–0.4]	0.905	0.2 (0.5) [0.1–0.3]
Handicap	0.2 (0.4) [0–0.3]	0.1 (0.5) [-0.1–0.4]	0.2 (0.6) [0–0.5]	0.877	0.2 (0.5) [0.1–0.3]
OHIP-14 physical	1.8 (1.8) [1.3–2.3]	2.4 (3.5) [0.7–4]	1.8 (2.6) [0.5–3]	0.556	1.9 (2.4) [1.4–2.4]
OHIP-14 psychological	0.7 (1.3) [0.4–1.1]	0.9 (1.3) [0.3–1.5]	1 (2.2) [0–2.1]	0.779	0.8 (1.5) [0.5–1.2]
OHIP-14 social	0.4 (0.9) [0.1–0.6]	0.3 (0.7) [0–0.6]	0.4 (1.1) [-0.1–1]	0.964	0.4 (0.9) [0.2–0.6]
**OHVS-12**					
Total	0.5 (0.1) [0.5–0.5]	0.5 (0.1) [0.5–0.5]	0.5 (0.1) [0.5–0.5]	0.979	0.5 (0.06) [0.5–0.51]
Professional dental care	0.3 (0.2) [0.2–0.3]	0.2 (0.2) [0.2–0.3]	0.2 (0.2) [0.1–0.3]	0.483	0.3 (0.2) [0.2–0.3]
Appearance and health	0.9 (0.1) [0.8–0.9]^*a*^	0.9 (0.1) [0.8–0.9]^*a*^	0.9 (0.1) [0.9–1.0]^*b*^	**0.034**	0.9 (0.1) [0.9–0.9]
Flossing factor	0.4 (0.1) [0.4–0.5]	0.4 (0.1) [0.4–0.5]	0.4 (0.1) [0.4–0.5]	0.761	0.4 (0.1) [0.4–0.5]
Retaining natural teeth	0.4 (0.2) [0.4–0.5]	0.5 (0.1) [0.4–0.5]	0.4 (0.1) [0.4–0.5]	0.428	0.5 (0.2) [0.4–0.5]

^*^Overall trend across age groups assessed by Kruskal–Wallis test; Different letters identify group differences detected by a post-hoc pairwise comparison. Values in bold indicate significant *p*-value.

Similarly, total OHVS-12 scores did not significantly differ between groups (*p* = 0.979), with a consistent mean score of 0.5 (±0.1) across all categories. Most OHVS-12 subdomains, such as professional dental care, flossing, and retaining natural teeth, also showed no significant group differences (Table 3).

However, a significant difference was identified in the “Appearance and Health” domain of the OHVS-12 (*p* = 0.034). Participants with periodontitis reported higher values in this dimension compared to healthy individuals, suggesting a greater self-perceived concern with appearance and oral health despite their clinical condition (Table 3).

PISA showed a significant positive correlation with PESA (rho = 0.660, *p* < 0.001), reflecting that the extent of inflammation increased with the epithelial surface area affected. Also, a significant positive weak correlation was observed between age and PISA (rho = 0.305, *p* = 0.003), indicating that older cadets tended to present with a greater inflamed periodontal surface area. No significant correlations were found between OHIP-14 total scores and age, PISA, or PESA. Likewise, OHVS-12 total score showed no relevant associations with any of the clinical or demographic variables. These results suggest that although periodontal inflammation appears to increase with age, it does not seem to influence self-perceived oral health impact or values in this population (Table 4).

**Table 4:** Correlation between age, PISA, PESA, OHIP-14, and OHVS-12 total scores, presented as Spearman's rank correlation coefficient (rho).

**Variables**	**PISA**	**PESA**	**OHIP-14 total**	**OHVS-12 total**
Age	**0.305**	0.149	−0.069	−0.007
PISA	–	**0.660**	−0.175	−0.006
PESA	–	–	−0.192	0.061
OHIP-14 total	–	–	–	−0.010
OHVS-12 total	–	–	–	–

Significant correlation coefficients denoted in bold (p ≤ 0.05).

## 4 Discussion

This study provides the first comprehensive characterization of periodontal health and psychosocial oral health indicators among PT-AF pilot cadets. Approximately 22.2% of the participants presented with periodontitis, and another 22.2% with gingivitis, highlighting that nearly half of this young, physically fit military population displayed signs of periodontal disease. These findings highlight that even within a highly selected and medically fit military population, periodontal inflammation can emerge early, underscoring the silent and progressive nature of this chronic condition. Clinically, this reinforces the importance of early periodontal screening and preventive care, as gingival inflammation is reversible when detected promptly. Integrating routine periodontal assessments into aviation medical protocols may reduce the risk of dental emergencies and help maintain dental fitness for flight and mission readiness.

When compared to other military populations, PT-AF cadets demonstrated a substantially higher prevalence of periodontal health (55.6%) than Belgian (24.3%) (15), Spanish (3.6%) (31), and U.S. counterparts (as low as 0% in some naval cohorts) (32, 33). The proportion of cadets with periodontitis (22.2%) was lower than other studies, and predominantly in the early stages of disease (Stage I–II, 11.1%), contrasting with higher rates and severity in Belgium (60.7% total; 54.2% early) and Spain (37.1% severe). Gingivitis prevalence (22.2%) was also lower than reported in U.S. studies (36.7–40.3%). Additionally, similar patterns of moderate periodontal burden have been reported in other military contexts. Among Indian Army male personnel, moderate periodontal involvement was frequent, and periodontal pockets and clinical attachment loss were associated with occupational stress and behavioral risk factors (34). Likewise, Finnish student military pilots recently demonstrated predominantly healthy periodontal conditions, with pilots falling exclusively within CPI 0–2 categories, indicating early periodontal changes despite being a highly selected and medically fit group (35). In Iranian soldiers, oral hygiene behaviors and overall oral status were shown to significantly influence periodontal health outcomes, again highlighting the interaction between lifestyle behaviors and the military working environment (36).

However, direct comparisons must be interpreted cautiously, as both diagnostic criteria and age structures varied markedly across studies.

In Belgium, Verhofstadt et al. (15) assessed 107 soldiers (mean age of 26 years) using the 2018 AAP/EFP case definition, reporting 55.1% mild and 6.5% moderate periodontitis. In Spain, Bárcena García et al. (31) applied the Community Periodontal Index of Treatment Needs (CPITN) in 229 military personnel (>19 years), finding 28% with periodontal pockets and 40.3% with attachment loss. In the U.S., Querna et al. (32) used an experimental periodontal index in 420 soldiers aged 18–24 years, detecting 27.4% early and 3.8% moderate or advanced periodontitis; Horning et al. (33), using similar criteria, found 30.0% early and 12.0% moderate or advanced forms among 697 military personnel aged 21–30 years. Additionally, Levin et al. (37) identified 5.9% aggressive periodontitis (4.3% localized, 1.6% generalized) in 642 Israeli recruits aged 18–30 years (mean 19.6).

Overall, these studies illustrate that diagnostic heterogeneity—ranging from CPITN to partial-mouth screening and experimental indices—and older mean ages (mid-20s to 30s) likely contribute to the higher reported prevalence and severity of periodontitis in previous military cohorts. In contrast, PT-AF cadets were examined using the 2018 AAP/EFP classification and full-mouth recordings at a mean age of 21.1 years, providing a more sensitive but age-restricted assessment of early disease. The comparatively lower prevalence of periodontitis and gingivitis in the present study may therefore reflect both the youth and health profile of the cadets and the adoption of standardized diagnostic protocols aligned with current international consensus, supporting the notion that early preventive monitoring can sustain periodontal health in high-performance military settings.

Regarding behavioral risk factors, smoking emerged as a key variable. Although 16.7% of cadets were current smokers, this prevalence is substantially lower than that reported in other European military populations, which ranges from 36% to over 59% (15, 38–40). It aligned with Portuguese civilian average (17.0%), reflecting a gradual cultural shift toward reduced tobacco use among younger adults (41). Similar rates were observed among Portuguese military personnel in previous studies, ranging from 31.7% (20) to 45.9%. Our findings are also comparable to those from U.S. Air Force recruits, with 9.8% current and 35.6% former smokers (17). These data reinforce the critical need for integrated smoking cessation strategies within military oral healthcare programs.

Oral hygiene habits among the cadets were overall satisfactory, with the majority brushing twice a day, mirroring patterns in both Portuguese military and civilian groups (42–44). However, only one-third practiced regular interproximal cleaning, a rate higher than in prior Portuguese military studies but still suboptimal. This finding suggests that while general oral hygiene awareness may be acceptable, specific preventive behaviors such as interdental cleaning are insufficiently incorporated into routine care. Such gaps highlight opportunities for targeted educational interventions emphasizing the importance of interdental plaque control.

Interestingly, despite the measurable presence of gingival inflammation and early periodontitis, the perceived impact on quality of live, as assessed by OHIP-14, was low. The OHVS-12 scores also revealed limited variability, with the exception of the “Appearance and Health” domain, which was higher in participants with periodontitis, perhaps reflecting increased concern with aesthetics once disease is recognized. This disconnection between clinical conditions and subjective perception may reflect limited awareness of disease consequences, a phenomenon well documented in young adult and military populations. Similar trends were observed in studies among Malaysian military personnel, where significant inflammation coexisted with low OHIP-14 score (45). In contrast, studies in New Zealand and Belgium reported that 18% of young recruits experienced significant oral health-related quality of life impacts (46, 47), similar to civilian data from Australia and the UK (48). While some studies report a disconnect between clinical and subjective measures, others show functional consequences, including sleep disruption and work impairment due to oral pain (49). These discrepancies suggest that psychosocial awareness of oral disease may depend heavily on cultural context, health literacy, and access to preventive services.

Moreover, the positive association between age and inflammatory burden, as measured by PISA and PESA, increased with age, even within this narrow age range of 18–25 years. The strong correlation between PISA and PESA suggests that tissue destruction progresses with disease severity, but this was not perceived as a burden by the cadets themselves. Importantly, these surrogate measures have been linked to systemic inflammatory load, endothelial dysfunction, and elevated cardiovascular risk markers in population-based studies (50–52). This reinforces the biological plausibility that even modest periodontal inflammatory surfaces can contribute to low-grade systemic inflammation (53, 54), which may be particularly relevant in high-performance contexts such as military aviation. The lack of self-reported symptoms despite measurable inflammation further support the need of objective periodontal surveillance and regular screening, as subjective measures alone may fail to capture early disease risk.

In Portugal, previous data have demonstrated that oral health literacy and preventive behavior among military personnel remain limited. Luis and Luis (55) revealed that only 54.7% of participants demonstrated adequate oral health literacy. Additionally, Azevedo et al. (20) reported that only 45.1% of military personnel had visited a dentist in the previous 6 months, while 18.9% had not had a dental visit in over a year. The main reasons for the low frequency of dental consultations included a perceived lack of need (43.4%), seeking care only in cases of pain (20.5%), and the cost of treatment (19.7%). These insights underline a crucial challenge, support the notion that, in this specific group, the clinical severity of periodontal disease does not correspond to participants' subjective perceptions of their oral health, nor does it significantly impact their reported quality of life. These findings can possibly be explained due to the lack of literacy about oral behaviors and consequences of periodontal disease among this type of population, devaluing, as already mentioned, these parameters. Furthermore, literature indicates that this concerning trend toward the devaluation of oral health in various military populations, characterized by low levels of oral health literacy lead to a tendency of only seeking dental care in emergency situations.

In this context, implementing structured oral health, behavioral reinforcement, and periodic screening programs within the Portuguese Air Force could improve both oral and systemic health outcomes. Beyond individual health benefits, maintaining optimal periodontal status is essential for operational readiness, as oral discomfort or infection can compromise nutrition, communication, and focus during flight missions.

### 4.1 Strengths and limitations

This study presents several methodological strengths. First, it included a representative sample of Portuguese Air Force pilot cadets, covering approximately 72% of all eligible cadets from the 1st to the 5th academic years, which reinforces internal validity and minimizes selection bias. The use of a single calibrated examiner ensured diagnostic consistency, and the application of the 2018 EFP/AAP case definition allows for comparability with other contemporary epidemiological studies. In addition, employing validated Portuguese versions of the OHIP-14 (24) and OHVS-12 (25) questionnaires provided a reliable assessment of participants' perceived oral health impact and values, thereby incorporating a patient-centered perspective rarely explored in military populations.

However, certain limitations should be acknowledged. The sample was racially homogeneous, as all participants identified as Caucasian, and predominantly male, reflecting the current demographic structure of the Portuguese Air Force. Although representative of this specific military cohort, such composition may limit the generalizability of findings to more diverse or civilian populations. The small number of female cadets prevented sex-stratified analysis, and future studies including more balanced samples are warranted. Additionally, although cadets begin flight-related activities in the 1st academic year, individual exposure to the aviation environment (e.g., flight hours or cumulative altitude exposure) could not be quantified, as such data were not available in the military academic registry. Recognizing this as an important occupational factor, future longitudinal studies should incorporate objective exposure metrics to evaluate potential dose–response associations between aviation conditions and periodontal outcomes. Furthermore, the cross-sectional design does not allow for causal inference, and longitudinal follow-up would be valuable to monitor periodontal changes throughout the training program.

Despite these limitations, this study provides novel and robust data on periodontal health and psychosocial indicators among Portuguese military air cadets. The findings support the inclusion of structured oral health surveillance and preventive strategies within occupational health programs in the military environment, and highlight the potential of integrating clinical and self-perceived oral health assessments to inform early, personalized preventive interventions.

## 5 Conclusion

This study provides the first comprehensive assessment of periodontal state among Portuguese Air Force pilot cadets, revealing a predominantly young male population with a notable presence of gingivitis and mild periodontitis. Despite low perceived impact on quality of life, clinical signs of inflammation and tissue destruction were present and associated with modifiable risk factors, including smoking habits use and suboptimal oral hygiene mainly in interdental care. These findings underscore the need to integrate structured periodontal screening and preventive strategies into military dental protocols. Given the specific demands of flight conditions, further epidemiological studies are recommended to deepen the understanding of oral health behaviors and needs within this population.

## Data Availability

The raw data supporting the conclusions of this article will be made available by the authors, without undue reservation.
